# Assessing Functional Communication in Persons With Aphasia: A Scoping Review of Formal and Informal Measures

**DOI:** 10.1111/1460-6984.70051

**Published:** 2025-05-10

**Authors:** Lauren Hammond, Thomas Christensen, Julius Fridriksson, Dirk B. den Ouden

**Affiliations:** ^1^ Arnold School of Public Health, Department of Communication Sciences and Disorders University of South Carolina Columbia USA

**Keywords:** aphasia, assessment, everyday communication, functional communication, life participation, nonverbal communication, quality of life, situated language use

## Abstract

**Background:**

The communicative effectiveness of persons with aphasia (PWA) has been assessed through a range of functional communication measures. However, variability in interpretations of what is covered by the term “functional communication” may have resulted in challenges to the implementation of appropriate and consistent patient‐centred evaluations, with different measures focusing on subsets of the components of functional communication.

**Aims:**

This paper aims to examine the current literature on informal and formal evaluation of functional communication in PWA and to identify gaps in currently available assessment tools.

**Methods:**

This scoping review included studies published between 1965 and 2024 that assessed functional communication in PWA, excluding studies focused on non‐aphasic populations or impairment‐based assessments without real‐world application. Systematic searches were conducted in PubMed, Embase, CINAHL, Scopus, and PsycINFO using predefined search terms. Of the 541 studies identified, 67 met the inclusion criteria after title/abstract and full‐text screening. Measures were categorized as formal (standardized) or informal (non‐standardized) and evaluated based on contextuality, multimodality, and interactiveness. Informal assessments also emphasized life participation, quality of life, augmentative alternative communication (AAC) strategies, conversational discourse, the informativeness and complexity of language use, and real‐world communicative transactions.

**Main Contribution:**

In the 67 studies included in the literature review, 32 functional communication assessments were identified across the categories of informal and formal evaluation. Informal assessments (28) included patient‐reported, clinician‐reported, observer‐reported, and performance‐based outcome measures. Formal functional communication assessments (4) included systematically normed instruments provided to PWA under controlled conditions, yielding a diagnosis or level of specified functional communication capability. Of the reviewed informal and formal measures, a limited quantity met all criteria for a comprehensive assessment of functional communication in aphasia, namely, being contextual, multimodal, and interactive.

**Conclusions:**

Existing assessments reveal gaps in the comprehensive evaluation of functional communication. The findings emphasize the need for standardized, multimodal, and context‐sensitive tools that better reflect the dynamic, real‐world communicative needs of PWA.

**WHAT THIS PAPER ADDS:**

*What is already known on the subject*

- Functional communication is recognized as a cornerstone for assessing real‐world abilities in persons with aphasia (PWA). However, current assessments vary in their design and implementation, reflecting the diverse approaches taken across clinical and research settings.

*What this paper adds to existing knowledge*

- This study emphasizes the importance of integrating contextuality, multimodality, and interactiveness into functional communication assessments. It highlights the valuable contributions of informal tools, which offer adaptability and focus on life participation, and identifies opportunities to enhance formal tools to better address real‐world communication needs.

*What are the potential or clinical implications of this work?*

- Developing standardized, holistic tools for functional communication assessment will provide clinicians and researchers with more effective resources to evaluate and support PWA. These advancements will promote consistency in practice, enable meaningful comparisons across studies, and ultimately improve life participation and communication outcomes for PWA.

## 1 Introduction

In an impairment‐oriented approach to aphasia, the field of speech language pathology promotes rehabilitation to improve areas of speech production, understanding, cognition, reading, and writing (Brookshire and McNeil 2014; Hallowell and Chapey 2008). As such, there has been a historical emphasis on the assessment and treatment of decontextualized linguistic units of language, as evidenced by foundational work (Duffy et al. 2008; Holland 1977). Aphasia diagnostic assessments traditionally prioritize isolated linguistic tasks, such as naming or sentence formation, often measured through accuracy scores or standardized assessments and typically generate scores that enable comparison across individuals (Goodglass et al. 1969). Examples of these assessments include subtests from the Western Aphasia Battery‐Revised (WAB‐R), the Boston Diagnostic Aphasia Examination (BDAE), and the Aphasia Diagnostic Profile (ADP); along with other assessments specifically designed for aphasia (Goodglass et al. 2001; Helm‐Estabrooks 1992; Kertesz 2007). Contemporary evidence suggests that these methods are insufficient in addressing real‐world communicative needs. Recent clinical practice guidelines and systematic reviews (Doedens and Meteyard 2020; Hinckley 2016; Simmons‐Mackie et al. 2016) draw attention to the importance of functional, context‐sensitive approaches that better align with the dynamic and interactive nature of real‐life communication. This brings to light the limitations of overreliance on decontextualized assessments for predicting and fostering real‐world communicative success.

Although standard decontextualized assessments are fairly inclusive of all major language domains, persons with aphasia (PWA) also experience communicative breakdown involving the exchange of personal information, ideas, feelings, and expression in day‐to‐day situations, which impacts their quality of life and life participation (Brumfitt 1993; Cruice et al. 2000; Hilari and Byng 2009). From other fields, such as psychology or sociolinguistics, comes a continued emphasis on the importance of contextualized communication in expressing personal identity, maintaining socioemotional relationships, and establishing shared ideas of personal concepts (Holmes 2005). Researchers often describe this as functional communication, which may be further defined as a one‐on‐one interaction evolving in actual time, between two or more individuals, that allows for multiple modes of communication and is grounded in the context provided by the present environment (Clark 1996; Forgas 2012; Gregory and Carroll 2018).

Restrictions in functional communication impact PWAs' ability to engage in everyday life activities. This reduced quality of communication limits their capacity to express intrinsic competence, share opinions, and convey internal thoughts, feelings, and emotions (Byng and Duchan 2005; Lyon and Shadden 2001; Parr et al. 1997). PWA face the challenge of relearning and re‐establishing the communicative connection with familiar and unfamiliar listeners across a multitude of real‐life scenarios.

**FIGURE 1:**
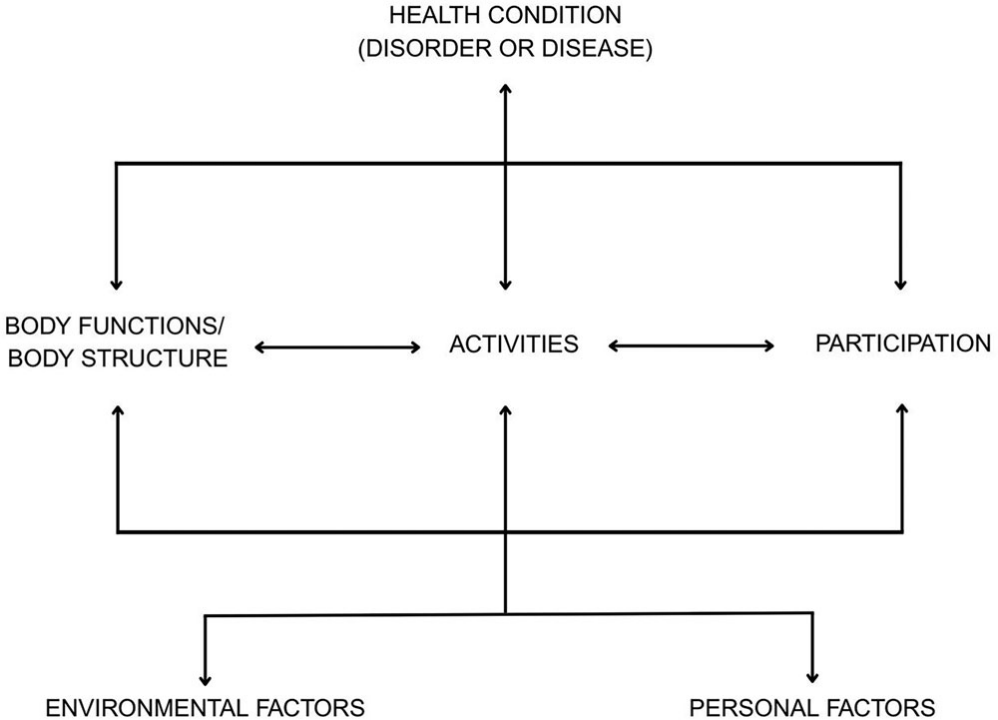
The international classification of functioning, disability and health to guide the selection of assessment methods (WHO 2001).

Functional communication is often referred to within the framework of the World Health Organization's International Classification of Functioning, Disability and Health (WHO‐ICF) as ‘communication activity limitation’ and ‘communication participation restriction,’ terms that emphasize the specific barriers individuals face in engaging in meaningful communication (Threats and Worrall 2004; Threats 2008; WHO 2001). This conceptualization aligns with the WHO‐ICF model (depicted in Figure 1), which illustrates how health conditions or disorders influence body functions and structures, activity limitations, and participation restrictions, all of which are further shaped by personal and environmental factors. Other terms used to describe functional communication in the existing literature include ‘situated language use’ (Tawil 2018), ‘environmental communication’ (Dalemans et al. 2010), ‘social conversation’ (Newhoff and Apel 1989), and ‘everyday communication’ (Harmon 2020). These partially interchangeable terms may reflect an existing challenge to the objective definition of ‘functional communication’.

The WHO‐ICF framework provides a multidimensional perspective on aphasia and functional communication by considering the dynamic interaction between physical, psychological, environmental, social, and assistive components (Simmons‐Mackie and Kagan 2007). Particularly, the ICF enables professionals to evaluate and address the diverse effects of aphasia. Likewise, this frameworks builds upon the interconnected aspects of community involvement and functional success. Additionally, principles set forth by the American Speech‐Language‐Hearing Association's (ASHA) Life Participation Approach to Aphasia (LPAA) have recognized the multifaceted communication and life‐involvement deficits experienced by PWA (Chapey et al. 2000). The LPAA and WHO‐ICF acknowledge that communication encompasses more than mere linguistic correctness, as it includes effective interaction within real‐world contexts.

Through the LPAA, collaboration between speech‐language pathologists (SLPs), clients, their families, and other professionals is encouraged, with the aim of reaching personally relevant communication objectives that are aligned with the individual's life roles and aspirations. This approach advocates for active participation and empowerment, permitting PWA to not only navigate their surroundings and express their needs but also to preserve their sense of self‐identity amid communication difficulties (Chapey et al. 2000; Holland 2007; Kagan 1998). By incorporating LPAA's emphasis on real‐world communication interactions, SLPs can gain a more accurate understanding of an individual's communicative abilities and limitations in everyday scenarios. Understanding how functional communication works in real‐life situations can help create personalized plans to help each PWA with their specific needs and goals. Such plans allow SLPs to provide more targeted and effective support strategies, recognizing that successful communication extends beyond language proficiency to encompass the individual's overall well‐being and participation in meaningful life roles.

Formal assessments are essential for achieving consistency and reliability, which in turn makes it easier to compare patient outcomes. Through standardization and common baselines, outcomes can be interpreted with a degree of accuracy across different contexts or time periods. This helps eliminate bias, ensures consistent evaluation, and facilitates meaningful comparisons, presumably leading to more reliable and valid outcomes. However, formal assessments often lack the ability to fully capture the individualized aspects of a person's abilities, experiences, or circumstances (Grosjean 1997; Worrall and Hickson 2008). Typically, they do not consider unique strengths, weaknesses, or contextual factors that can influence performance. By contrast informal assessments offer more flexibility for SLPs during administration and may shift focus from impairments to abilities in the assessment of communicative success. An informal assessment is a non‐standardized evaluation method used to observe an individual's abilities and challenges in naturalistic settings, often focusing on practical, functional ability, rather than structured testing (Brookshire and McNeil 2014; Hegde and Kuyujian 2019). However, recognition of the limitations of isolated informal measurements has fed a demand for more concrete quantification of functional communication proficiency. Without a universal, multimodal, contextual, and interactive functional communication measure, the field of aphasiology will remain limited in its research and clinical assessment of the ability of PWA to communicate in the natural world (Doedens and Meteyard 2020; Hartley 1990).

While the assessment of functional communication abilities can be subjective and largely observational, both formal and informal assessment methods exist, originating from a range of perspectives on ‘what it is’ that should be measured (Aldridge et al. 2023; Wallace et al. 2022). Wallace and colleagues identified various measurement instruments used for PWA. Their scoping review focused on instruments targeting different domains such as language, cognition, and psychosocial aspects, exploring their utility and relevance in clinical practice. The review highlighted gaps in the availability of holistic and accessible tools and emphasized the need for more individualized, context‐specific assessments (Wallace et al. 2022). In another scoping review, Aldridge et al. (2023) focused on identifying and examining tools used to assess functional communication in individuals with acquired communication disorders, particularly in the context of real‐life communication challenges. While many tools are performance‐based and designed for specific clinical populations, only a few consider the multimodal aspects of communication or are adaptable for individual needs (Aldridge et al. 2023). Expanding on this prior research, the current scoping review will extend the discussion by concentrating on the practical application of these assessments in real‐world settings, going beyond the identification of these tools and aiming to assess how effectively they capture dynamic, real‐time communication in everyday situations. Additionally, we focus on multimodal communication including gestures and augmentative alternative communication (AAC).

Despite growing momentum toward functional paradigms and a broader understanding of health and individualized care, the integration of functional communication assessments into research and clinical practice remains inconsistent. While recent large‐scale international research initiatives, such as the RELEASE trial (Brady et al. 2022), have successfully incorporated functional communication measures, they also highlight the need for further development and refinement of assessment tools to ensure widespread application. Notably, the Core Outcome Set for Aphasia Research (ROMA‐2) emphasizes the importance of functional communication assessments in capturing real‐world communicative abilities in PWA (Wallace et al. 2023). The ROMA‐2 initiative identified a need for greater alignment between assessment tools and the dynamic, multimodal, and interactive nature of everyday communication. However, challenges remain that may limit consistent integration. These include the historical emphasis on linguistic models, reimbursement and policy structures, lack of training and comfort when addressing functional communication, challenges in measuring functional communication, systemic constraints, evidence gaps, and cultural resistance to change (Blonski et al. 2014). While recent progress indicates a shift in focus (Aldridge et al. 2023; Doedens and Meteyard 2020; Harmon 2020; Meteyard 2020; Milman and Murray 2024; Wallace et al. 2022, 2023), the identification of these barriers reinforces the critical need for the development of more standardized, multimodal, and context‐sensitive tools. Such efforts would strengthen the clinical utility and impact of functional‐communication assessments in enhancing life‐participation outcomes for PWA.

How do we qualitatively and quantitatively capture and value the ability to achieve communicative goals, arguably the most important aspect of an individual's behavioural assessment, for the selection of intervention approaches and the evaluation of progress and response to treatment? While it is tempting to view language and functional communication as synonymous, they are in fact separate concepts, even if highly related (Holland and Hinckley 2002; Kagan and Simmons‐Mackie 2007; Threats and Worrall 2004; Worrall and Hickson 2008). Doedens and Meteyard define “language” as the use of decontextualized linguistic units in isolation and “functional communication” as *contextualized*, *multimodal*, and *interactive* (Doedens and Meteyard 2020). For the formulation of a clinically and methodologically useful definition of ‘functional communication’, it is important that these concepts are defined.

Contextuality highlights the importance of adapting communication strategies to different real‐world situations, considering the environment, culture, and the relationship between communicators. Multimodality recognizes that communication is not limited to spoken language but also includes gestures, facial expressions, and body language, allowing individuals to express meaning in various ways. Interactiveness focuses on the dynamic, reciprocal nature of communication, where individuals engage in real‐time exchanges, adjusting their messages based on feedback from others. Functional communication abilities of PWA rely on individual strengths and weaknesses across multimodal communication and are dependent on factors such as the person's access to intervention and ability to utilize AAC where helpful. The interactive adaptation of communication to specific situations as well as communication partners is crucial for effective information exchange (Kagan and Simmons‐Mackie 2007).

To effectively assess functional communication, we must first understand the foundational skills that are required to communicate in everyday life. Communication is rooted in context, subjectivity, and uniqueness to the person (Anand and Korotkova 2022; Berger 2014; Blumer 1986; Carter and Fuller 2015). For a functional communication measure to be maximally naturalistic, it should be minimally constrained and must allow the participant full access across modalities. Although it is generally accepted that situated language use requires multi‐level communicative processes, there are clinical and research challenges that impede the ability to formally assess the comprehensive nature of functional communication (Worrall and Hickson 2008). Drawing from Doedens and Meteyard's emphasis on *contextuality*, *multimodality*, and *interactiveness*, this review aims to outline existing available assessments of functional communication that adhere to these criteria. Furthermore, we identify potential directions for future development, with the ultimate goal of promoting improved life participation for PWA.

### 1.1 Aims

The primary aim of this study is to provide a scoping review of existing functional communication assessments for PWA. The objective is to provide SLPs and researchers with essential information on the tools available to evaluate functional communication, focusing on their alignment with real‐world communicative needs. For this purpose, functional communication assessments are defined as tools, both formal and informal, designed to evaluate communication skills through the lenses of contextuality, multimodality, and interactiveness, ensuring that the assessment takes place within meaningful, context‐driven environments that facilitate varied modes of communication and active participant engagement.

The guiding research questions were as follows:

- What functional communication assessments are currently available for PWA?
- How do these tools incorporate the principles of contextuality, multimodality, and interactiveness?
- What shared features emerge across informal assessments, particularly regarding their focus on life participation and quality of life, use of AAC, salient conversational discourse, the complexity of language, and naturalistic scenarios?
- What limitations exist in current functional communication assessments?
- What potential directions can be proposed for the development of novel functional communication assessments to address these limitations and improve clinical utility?

## 2 Methods

A scoping review of the literature was conducted to identify assessments that investigated functional communication in PWA. We utilized Covidence, an online tool designed for managing systematic reviews, to facilitate and streamline the review process (Veritas Health Innovation 2023). This paper adheres to the reporting guidelines outlined in the PRISMA Scoping Review Extension (Tricco et al. 2018).

### 2.1 Database Search to Identify Relevant Studies

In December 2024, we conducted searches across five databases: CINAHL, EMBASE, PsycINFO, Scopus, and PubMed. To ensure a broad capture of relevant literature, Google Scholar was also utilized to supplement the review as part of the process of identifying additional records through reference lists. The search terms were carefully designed to capture the clinical populations of interest and account for the variability in terminology used to describe the assessment of functional communication. This process included assessments referenced in Doedens and Meteyard (2020). A total of 30 search terms related to functional communication in PWA are outlined in Table 1.

**TABLE 1:** Database search terms.

Population	Communication profile	Task	Focus
*stroke* ***OR*** *traumatic brain injury* ***OR*** *acquired brain injury*	*Aphasia*	*functional* ***OR*** *effective* ***OR*** *meaningful* ***OR*** *practical* ***OR*** *conversation* ***OR*** *exchange* ***OR*** *interaction* ***OR*** *discourse* ***OR*** *dialogue* ***OR*** *interactive* ***OR*** *reciprocal* ***OR*** *dynamic* ***OR*** *collaborative* ***OR*** *multimodal* ***OR*** *gestural* ***OR*** *augmentative* ***OR*** *alternative* ***OR*** *integrated* ***OR*** *contextual* ***OR*** *environmental* ***OR*** *situated* ***OR*** *social*	*assessment* ***OR*** *outcome measure* ***OR*** *evaluation* ***OR*** *quantification*

### 2.2 Eligibility Criteria

Studies were considered eligible if they met the following criteria:

- Participants were adults with aphasia resulting from stroke or brain injury. Studies involving non‐aphasia populations or paediatric populations were excluded.
- The focus of the study was on functional communication assessed within real‐world settings or contexts. Studies focusing solely on impairment‐based measures without real‐world applicability were excluded.
- The assessment tools evaluated were formal or informal and incorporated multimodal, contextual, and interactive aspects of communication. Tools focusing exclusively on linguistic components, such as grammar or naming, were excluded.
- Studies with an intervention focus but lacking functional communication outcomes were excluded.
- Studies published between 1965 and 2024 were included, while those published before 1965 were excluded.
- Only studies available in English were considered; non‐English studies were excluded.

### 2.3 Data Extraction and Charting

**FIGURE 2:**
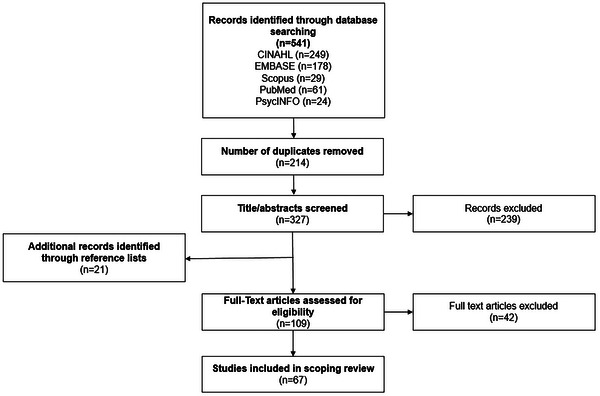
PRISMA flow diagram of the selection of sources of evidence.

The search results were imported into Covidence (Veritas Health Innovation 2023), where duplicate records were identified and removed using both manual and automated methods. A PRISMA flow diagram (Figure 2) illustrates this process. To pilot the eligibility criteria, two authors (LH and TC) independently reviewed an initial set of 40 papers, achieving 82% agreement. Their comparisons prompted minor adjustments to the criteria, such as excluding studies involving individuals with dementia, primary progressive aphasia or other communication disorders and refining the definition of functional communication. During the title and abstract screening, 50% of citations were reviewed by these two authors independently, resulting in 85% agreement. Similarly, 25% of citations underwent double screening during the full‐text review stage, achieving 95% agreement. Any discrepancies in judgments were resolved through discussion and consensus. In cases where agreement was not initially reached, a third review was conducted, and decisions were made based on a final consensus. By applying these standards, we ensured that the assessments included in this review were judged on how well they captured the dynamic, multifaceted nature of functional communication in PWA.

The initial data extraction template was developed by the lead author (LH) based on the research questions guiding this scoping review on functional communication assessments for PWA. The template was piloted and collaboratively refined by two authors (LH and TC) to ensure it aligned with the review's objectives (Table 2). Data were systematically extracted to provide a detailed description of the studies included. This process captured key contextual details, including the clinical population (e.g., stroke, TBI, or acquired brain injury) and participant characteristics (e.g., age, diagnosis of aphasia). Information specific to the assessments was also recorded, such as the name and type of assessment (formal or informal), outcome measure classification (e.g., patient‐reported, clinician‐reported), and criteria evaluated (contextuality, multimodality, and interactiveness). Task‐related details, including the description of the activity and assessment setting, were also extracted. This structured approach ensured that the extracted data provided a comprehensive and systematic foundation for analysing the identified assessments, addressing the review's core research questions, and synthesizing findings.

**TABLE 2:** Data extraction guideline.

Study	Participants	Assessment	Criteria
Author(s) Year of publication Aim of the study Population	Demographic Aphasia type and severity	Name and type Outcome measure classification Delivery mode Validity and reliability	Description of task/activity Assessment setting Contextual, Multimodal, and/or interactive

### 2.4 Formal and Informal Assessment Categorization

Functional communication assessments found in the literature were categorized as either formal or informal, based on definitions supported by established texts (Fridriksson et al. 2022; Shipley 2023). Formal assessments were defined as standardized tools that include both norm‐referenced and criterion‐referenced measures. These assessments are consistently administered and scored and have undergone rigorous psychometric evaluation to ensure reliability and validity. Norm‐referenced assessments compare an individual's performance to a normative sample, while criterion‐referenced assessments evaluate performance against predefined benchmarks or skill sets, focusing on mastery of specific abilities.

In contrast, informal assessments were defined as non‐standardized tools designed to provide qualitative, observational data that are flexible and tailored to the individual's communication needs in natural contexts. Informal assessments are typically used to gather insights into functional communication behaviours without direct comparison to normative data or specific benchmarks. These tools are particularly valuable for understanding real‐world communicative success and identifying individualized intervention goals.

### 2.5 Contextual, Multimodal, and Interactive Criteria

In categorizing the functional communication assessments identified from the literature, we applied three key criteria—contextuality, multimodality, and interactiveness (Doedens and Meteyard 2020)—to evaluate the comprehensiveness of each tool. These criteria were chosen to reflect the real‐world, practical nature of functional communication, where PWA must communicate using various modes of expression, across multiple contexts, and through interactive exchanges.

An assessment was considered contextual if it evaluated communication within real‐world, practical, or everyday settings. Assessments needed to involve communication tasks that were grounded in real‐life scenarios (e.g., social interactions, using the phone, asking for help) or that took environmental factors into account. To meet this standard, the assessment had to simulate or directly assess communication within functional, situated contexts, beyond clinical or artificial settings.

To qualify as multimodal, an assessment needed to incorporate or account for more than one mode of communication. This could include verbal communication, non‐verbal communication (e.g., gestures, facial expressions), as well as AAC strategies. Assessments that allowed for or explicitly measured the integration of these multiple communication modes were categorized as multimodal.

An assessment was categorized as interactive if it involved dynamic, two‐way communication between participants or between a participant and an examiner. This criterion required the assessment to capture reciprocal, real‐time exchanges, where both participants actively responded and adjusted their communication based on feedback. Assessments focusing on conversational turn‐taking, dialogue, or social exchanges were marked as interactive.

Each assessment was categorized based on whether it met one, two, or all three of these criteria. Assessments that evaluated communication in real‐life settings, incorporated multiple communication modes, and involved interactive exchanges between individuals were categorized as fulfilling all three standards. If an assessment only evaluated communication in context but did not incorporate multimodal communication or interactive exchanges, it was categorized accordingly. To ensure reliability in categorization, two independent raters (authors LH and TC) reviewed each assessment. Both raters applied the same criteria to determine whether the assessments met the standards for contextuality, multimodality, and interactiveness. Any discrepancies in judgments were resolved through discussion and consensus. In cases where agreement was not initially reached, a third review was conducted, and decisions were made based on a final consensus. By applying these standards, we ensured that the assessments included in this review were judged on how well they captured the dynamic, multifaceted nature of functional communication in PWA.

In the exploration of functional communication assessments, some widely used tools were excluded from the primary review due to their broader applicability across multiple communication disorders, not specifically focusing on aphasia. These assessments do not specifically address challenges faced by PWA and are therefore less relevant in the context of an aphasia‐specific review. However, it is important to recognize their utility in measuring communication outcomes in diverse clinical populations. Therefore, we chose to list and discuss such measures separately while limiting the primary analyses and discussion to aphasia‐specific measures.

## 3 Results

The 34 assessments we identified address key aspects of functional communication, including its use in context, its multimodal nature, and its interactive application. Few of the assessments involve all three of these components, and no assessment that involved all three criteria was standardized. Although some components may be implicitly involved within the assessments, Table 3 lists only the components that are explicitly assessed. Each criterion is marked as Yes, No, or Limited to reflect its degree of inclusion in the assessment. A Yes indicates that the criterion is explicitly and comprehensively addressed, while a No signifies that the criterion is absent from the assessment. Limited denotes partial inclusion, where the criterion is addressed to some extent but is not the primary focus or is inconsistently evaluated. These designations ensure transparency in evaluating the scope and comprehensiveness of each assessment.

**TABLE 3:** Functional communication assessments and classification types.

Assessment	CT	C	M	I	Reference
**Formal (standardized)**					
Amsterdam‐Nijmegen Everyday Language Test (ANELT)	PB	Yes	No	Yes	Blomert et al. (1994)
Communication Activities of Daily Living (CADL‐3)	PB	Yes	Yes	Limited	Holland et al. (2018)
The Scenario Test (TST)	PB	Yes	Yes	Limited	van der Meulen et al. (2010)
ASHA Functional Assessment of Communication Skills (ASHA‐FACS)	CR	Yes	Yes	Limited	Frattali et al. (1995)
**Informal (non‐standardized)**					
Therapy Outcome Measure (TOM)	CR	No	Limited	No	Enderby et al. (2013)
Functional Communication Profile (FCP)	CR	No	No	No	Sarno (1969)
Communicative Effectiveness Index (CETI)	OR	Yes	Limited	Yes	Lomas et al. (1989)
Functional Outcome Questionnaire for Aphasia (FOQ‐A)	OR	Yes	No	No	Ketterson et al. (2008)
Communication Activity Log (CAL)	OR	Yes	No	No	PulvermüLler et al. (2001)
Aphasia Communication Outcome Measure (ACOM)	PR	Yes	Yes	Yes	Doyle et al. (2012)
Communication Outcome After Stroke (COAST)	PR	Yes	No	Yes	Long et al. (2008)
Aphasia Impact Questionnaire (AIQ‐21)	PR	Yes	Limited	Limited	Swinburn et al. (2019)
Revised Edinburgh Functional Communication Profile (REFCP)	CR	Yes	No	Yes	Wirz et al. (1990)
Inpatient Functional Communication Interview (IFCI)	CR	Yes	Limited	Yes	O'Halloran et al. (2019)
Social Networks: Communication Inventory	OR	Limited	No	Yes	Blackstone and HuntBerg (2003)
Verbal Activity Log (VAL)	OR	No	No	No	Johnson et al. (2014)
Communication Participation Item Bank (CPIB)	PR	Yes	Limited	Yes	Baylor et al. (2013)
Transactional Success (TS)	OR	Yes	Yes	Yes	Ramsberger and Rende (2002)
Frenchay Aphasia Screen Test (FAST)	CR	No	No	No	Enderby et al. (1987)
Assessment of Communicative Effectiveness in Severe Aphasia (ACESA)	PB	Yes	Yes	No	Cunningham et al. (1995)
Multimodal Communication Screening Test (MCST)	OR	Yes	Yes	No	Lasker and Garrett (2006)
Conversational Analysis (CA)	PB	Yes	No	Yes	Beeke et al. (2007)
Correct Information Units (CIU)	PB	No	No	Yes	Nicholas and Brookshire (1993)
Informational Units (IU)	PB	No	No	Yes	McNeil et al. (2001)
AAC Categories of Communicators Checklist	OR	Yes	Yes	No	Garrett and Lasker (2009)
Aphasia Needs Assessment (ANA)	PR	No	No	Limited	Garrett and Beukelman (2006)
The Speech Questionnaire (TSQ)	CR	Limited	No	Limited	Lincoln (1982)
Measure of the Participation in Conversation (MPC)	CR	Yes	Limited	Yes	Kagan et al. (2004)
The Communication Confidence Rating Scale for Aphasia (CCRSA)	PR	Yes	Limited	No	Babbitt et al. (2011)
Assessment for Living with Aphasia (ALA)	PR	Yes	Yes	Limited	Kagan et al. (2010)
The Communicative Activities Checklist (COMACT)	PR	Yes	Limited	Limited	Cruice (2002)
The Social Activities Checklist (SOCACT)	PR	Yes	No	Limited	Cruice (2012)

Abbreviations: C = contextual; CR = clinician‐reported; CT = classification type; I = interactive; M = multimodal; OR = observer‐reported; PB = performance‐based; PR = patient‐reported.

### 3.1 Formal Assessments

In this review of 67 relevant functional communication studies, 4 standardized assessments were identified as widely available and designed for easy administration by SLPs. These norm‐referenced and criterion‐referenced evaluations demonstrate probing of communicative markers in PWA across real‐life situations. To assess the comprehensiveness of functional communication assessments, we examined their critique of the use of language in context, multimodal communication, and interactive aspects of situational adherence.

#### 3.1.1 Amsterdam‐Nijmegen Everyday Language Test (ANELT)

The ability to communicate effectively beyond familiar scenarios while using a variety of modes is a crucial component of functional communication. The ANELT is a standardized functional communication measure normed on PWA that auditorily provides a series of real‐life scenarios (Blomert et al. 1994). It consists of 10 items designed to depict everyday situations, which are presented in a script‐like format without visual depiction of the auditory context. The participants' verbal communication is assessed by scoring understandability and intelligibility using a five‐point scale, along with evaluating its subcomponents. Although this formal measure provides insight into the verbal effectiveness and efficiency of persons with mild aphasia, it does not assess corresponding modes of communication alongside the verbal modality. This tool assesses whether the content of the message given by the client is interpretable and scores intelligibility. Nonverbal communication is only scored if it is used alongside verbal communication. Communicative ability is measured using dialogue‐structured scenarios with tangible items to support auditory prompts, such as a stained shirt, a damaged shoe, and a pair of gloves. Without discernment of a speaker's ability to successfully use multimodal communication, scores reported from the ANELT may serve as unreliable for the identification of a significant deficit in functional communication, or lack thereof. Even though each mode of communication functions interdependently, each contributes to the formation of successful communication transactions, critical for the assessment of functional communication ability (Ramsberger and Rende 2002).

The ANELT was specifically designed to reflect real‐life communication scenarios, making contextuality one of its key features. The assessment focuses on how PWA perform in practical, everyday settings, such as role‐playing conversations with a doctor or discussing plans. By simulating these real‐world situations, the ANELT evaluates functional communication within meaningful contexts. Another important aspect of the ANELT is its interactiveness. The test requires active, conversational exchanges, where PWA interact with an examiner or another person during role‐playing scenarios. These interactions emphasize back‐and‐forth communication, as the PWA is tasked with responding to questions, making requests, or carrying out conversations, ensuring that the interactive nature of communication is central to the assessment. However, multimodality is not a major focus of the ANELT. While the test assesses verbal communication in everyday scenarios, it does not explicitly prioritize or evaluate non‐verbal modes of communication, such as gestures or AAC, in the way some other assessments do. The ANELT's primary emphasis is on verbal exchanges within specific contexts and the interaction between individuals, rather than on the use of multiple communication modalities.

#### 3.1.2 Communication Activities of Daily Living (CADL‐3)

The Communication Activities of Daily Living (CADL‐3) is a formal functional communication measure that assesses diverse modes of communication across reading, writing, nonverbal communication, and social transactions with given visual and verbal picture‐based scenarios (Holland et al. 2018). This 50‐item assessment was normed on adults presenting with aphasia following a cerebrovascular accident, traumatic brain injury, tumour, or other brain insults. Specifically, the CADL‐3 assesses communication activities in seven areas: reading, writing, and using numbers; social interactions; contextual communication; nonverbal communication; sequential relationships; humour, metaphor, and absurdity; and internet basics through a stimulus book of photographs with items pertaining to the basic use of technology including mobile phones, the internet, and email. While the CADL‐3 evaluates a broad spectrum of communication skills, it could be challenging to gauge salient communicative skills using non‐interactive photographs that cannot be adjusted according to the ability level within the given situation. In addition, the assessment specifically targets and measures the unidirectional transmission of a message, without interpreting the interactive aspect of communication (Holland et al. 2018). With the focus on a linear model of communication, the CADL‐3 does not assess PWA in a ‘collaborative’ or ‘transactional’ model of communication. Real‐life communicative situations are dependent on a speaker transmitting a message to the listener, the listener comprehending the message, and the listener then becoming the speaker, interactively.

The primary focus of the CADL‐3 remains on real‐world relevance, ensuring that the communication skills assessed are directly applicable to practical, daily tasks. This focus on contextuality ensures that the CADL‐3 measures a person's ability to navigate common communication situations encountered in daily life. The CADL‐3 also values multimodality, allowing individuals to demonstrate their communication skills through various channels. This includes verbal, non‐verbal, and written communication, recognizing that many individuals, especially those with communication impairments, rely on multiple modes to effectively communicate in different scenarios. Although the CADL‐3 includes situations that involve interaction, such as responding to questions or engaging in a phone call, the tool places greater importance on assessing how individuals manage real‐world communication tasks. The dynamic, back‐and‐forth nature of a conversation, while present in certain items, is not the primary focus of the CADL‐3. Therefore, interactiveness is less of a central feature in this test, relative to its focus on contextuality and multimodality.

#### 3.1.3 The Scenario Test

The Scenario Test is a standardized measure and normed on PWA in the United Kingdom. This realistic functional communication measure quantifies the communicative effectiveness of conversational attempts and provides a score for the communicative ability of the PWA in an interactive setting (van der Meulen et al. 2010). The stimuli are provided via visually drawn pictures paired with interactive narratives. The Scenario Test is scored on speaking or writing, non‐verbal communication such as gestures, drawing, or using a communication device, level of facilitative prompting needed, and communicative effectiveness within verbal and non‐verbal communication modalities. It presents six everyday scenarios and asks the PWA to adopt the role of a character who is faced with a communicative task with a ring‐bound book of illustrations.

The test was designed for adults diagnosed with severe aphasia who express no or very limited verbal language, and van der Meulen et al. (2010) specifically validated it for this population. Its applicability to individuals with mild to moderate aphasia has not been systematically evaluated. Therefore, while it is primarily used with individuals with severe aphasia, further research is needed to determine its suitability for those with less severe impairments.

The primary goal of the Scenario Test is to assess how PWA manage the communication challenges they face in their daily lives. Due to its strong emphasis on real‐world scenarios, contextuality is a central element of the test, ensuring that it closely mirrors the communication demands of everyday situations. Additionally, the Scenario Test places a significant emphasis on multimodality, encouraging participants to use a variety of communication methods to make themselves understood. Individuals are allowed to use gestures, writing, pictures, or other alternative forms of communication to complete tasks, demonstrating the importance of multiple communication modes. This focus on multimodal communication ensures that participants can utilize a wide range of strategies to convey meaning. While the Scenario Test assesses communication in social contexts, it does not place as much emphasis on the conversational turn‐taking aspect of communication, which is a hallmark of interactiveness. Instead, the test primarily focuses on how individuals use different communication modes to express themselves in real‐world scenarios. As a result, interactiveness is less prominent in this assessment compared to its focus on contextuality and multimodality.

#### 3.1.4 American Speech‐Language‐Hearing Association Functional Assessment of Communication Skills for Adults (ASHA FACS)

The ASHA FACS is a standardized assessment that evaluates how well adults with communication disorders perform in real‐life situations. It focuses on six areas: social communication, communication of basic needs, reading, writing, number concepts, and daily planning (Frattali et al. 1995; Paul 2017). This tool is commonly used for PWA, traumatic brain injury, or other communication disorders. ASHA FACS allows for the assessment of various communication modalities, including verbal and non‐verbal communication (e.g., gestures, facial expressions, and body language). This makes it relevant for individuals who use a combination of communication strategies to express themselves. The assessment is highly contextual, as it evaluates functional communication in everyday situations. It assesses how individuals communicate in meaningful, real‐world contexts, such as interacting socially, meeting basic needs, and planning daily activities. While the ASHA FACS evaluates interactive communication by assessing how individuals manage conversations and social exchanges, its level of interactivity is limited. The clinician‐rated nature of the assessment relies on observations rather than live, dynamic exchanges, which may miss the complexity of real‐time conversational adjustments. Additionally, while it incorporates both verbal and non‐verbal communication, it may not fully capture the subtleties of more dynamic or collaborative interactions, such as turn‐taking in extended dialogues or the integration of AAC strategies in interactive contexts. This focus restricts its ability to assess deeper levels of interactivity that are essential in more naturalistic communication settings.

### 3.2 Informal Assessments

In reviewing 28 informal assessments of functional communication, we identified two that met the key criteria of contextuality, multimodality, and interactiveness. These assessments comprehensively evaluate communication within real‐life contexts, the use of multiple communication modes, and the interactive nature of conversational exchanges. In addition to the key criteria, several other key trends were observed across our informal assessments. These include a focus on life participation and quality of life, the use of AAC strategies, the assessment of salient conversational discourse, the examination of communicative transactions during real‐life scenarios, and the evaluation of the informativeness and complexity of language use. Each of these elements highlights different dimensions of functional communication, tailored to the specific research or clinical objectives behind the development of each assessment.

#### 3.2.1 Aphasia Communication Outcome Measure (ACOM)

The ACOM is a self‐reported outcome measure used to evaluate the functional communication abilities of PWA. It assesses the impact of aphasia on communication in everyday life and tracks changes over time, based on the individual's perceived communication effectiveness (Doyle et al. 2012). The ACOM considers the use of multiple communication modalities, including both verbal and non‐verbal forms of communication, as PWA often rely on alternative methods such as gestures, facial expressions, and other non‐verbal cues to communicate. The self‐report format captures the individual's communication performance in everyday social and practical settings, making it closely tied to functional, real‐world communication. This assessment also incorporates interaction by assessing how individuals engage in dialogue and social exchanges. It evaluates the extent to which a PWA can successfully navigate back‐and‐forth communication in daily conversations, including how well they can respond to others and maintain a conversational flow. The ACOM is a self‐report measure, yet individuals may have difficulty accurately assessing their own communication abilities, particularly if they have co‐occurring cognitive or memory impairments. This can result in inconsistent or inaccurate responses. The subjective nature of self‐report tools can limit the objectivity of the data, as PWA may overestimate or underestimate their communication abilities based on their confidence or emotional state. Research has shown that self‐reported communication effectiveness does not always align with observed performance, particularly in individuals with communication disorders (Richardson et al. 2016; Wallace et al. 2023). The ACOM focuses on perceived communication effectiveness, which may not fully correlate with an individual's actual communication performance in real‐world situations (Babbitt et al. 2011). Additionally, self‐report measures are susceptible to biases such as social desirability and response bias, which can influence responses and limit the accuracy of the data (Podsakoff et al. 2003). As a result, relying solely on self‐reported communication ability may leave gaps in understanding the extent of functional deficits

#### 3.2.2 Transactional Success

The Transactional Success assessment measures the effectiveness of communication between individuals in goal‐oriented interactions. It focuses on how successfully communication partners can achieve their intended outcomes during specific tasks, such as purchasing an item or giving directions (Ramsberger and Rende 2002). Transactional Success features multiple communication modes, such as spoken language, gestures, and the use of communication devices (when necessary), allowing individuals to communicate through whatever means they find most effective in order to complete a transaction. This assessment is inherently contextual, as it examines communication in real‐world tasks that people perform regularly. It evaluates how well individuals can successfully communicate in everyday transactions, such as making a purchase or asking for directions, with an emphasis on accomplishing the intended outcome. Interaction is a core component of this assessment, as it focuses on communicative success during two‐way exchanges with another person. The transactional nature of these interactions requires the individual to engage in back‐and‐forth communication to ensure that both parties understand each other and achieve their goals.

The focus on goal‐oriented tasks limits the types of communication assessed by Translational Success. The measure is not designed to evaluate social conversations or emotional exchanges, which are also crucial for functional communication. Since the primary focus is on achieving the transactional goal, the assessment may not fully capture the quality of the interaction or the depth of conversational engagement, particularly in more complex social exchanges. Transactional interactions often involve structured, predictable tasks, which may not fully generalize to more spontaneous or unpredictable social interactions in everyday life.

### 3.3 Shared Features

Beyond the primary criteria, our informal assessments revealed several other notable trends, including a focus on life participation and quality of life, the use of AAC strategies, the evaluation of salient conversational discourse, the assessment of the informativeness and complexity of language use, and the analysis of communicative transactions in real‐life scenarios.

#### 3.3.1 Quality of Life and Life Participation

Interviews, family or caregiver surveys, and self‐report measures capture a comprehensive view of an individual's communication‐related quality of life and life participation. This review focuses on assessments that measure functional communication while also addressing aspects of quality of life and/or life participation, rather than those that exclusively assess quality of life. Accordingly, included assessments are the Aphasia Impact Questionnaire (Swinburn et al. 2019), ACOM (Doyle et al. 2012; Hula et al. 2015), Communication Activity Log (PulvermüLler et al. 2001), Functional Outcome Questionnaire (Ketterson et al. 2008), and Communication Outcomes After Stroke (Long et al. 2008; Long et al. 2009). These assessments support the enhancement of the overall well‐being and participation of PWA in society by providing valuable feedback to the individual and caregivers and creating awareness of preserved communicative ability. They emphasize contextuality by evaluating communication within the personal and social spheres of PWA. These tools assess how effectively PWA convey and comprehend information in real‐world situations, reflecting on the impact of communication on their quality of life and societal participation. By focusing on the individual's day‐to‐day environment, these assessments provide insights into the subjective experience of PWA, aiding in the enhancement of their communicative function and overall well‐being.

#### 3.3.2 Use of Augmentative and Alternative Communication (AAC)

By thoroughly assessing AAC use in PWA, SLPs can better understand unique profiles of communication challenges, which helps to design personalized AAC systems or strategies that enhance communication. The informal assessments that evaluated the use of AAC included the Aphasia Needs Assessment (Garrett and Beukelman 2006), AAC Categories of Communicators Checklist (Garrett and Lasker 2009), and the Multimodal Communication Screening Test (Lasker and Garrett 2006). These assessments highlight accessibility to communication support, adaptable preferences, and overall functional communication needs. Additionally, these assessments ascertain the multimodal nature of communication, considering various modes and forms of expression to tailor communication strategies that suit the individual's unique requirements.

#### 3.3.3 Salient Conversational Discourse

Salient conversational discourse in PWA is crucial for improving real‐life communication proficiency, enhancing functional relevance, and identifying communication breakdown during personal dialogue. Measures that assess this criterion include the Verbal Activity Log (Johnson et al. 2014), the Measure of the Participation in Conversation (Kagan et al. 2004), and Conversational Analysis (Beeke et al. 2007). Conversational Analysis emphasizes interactiveness, looking at how individuals respond to one another, manage turn‐taking, repair misunderstandings, and use conversational cues. The Measure of the Participation in Conversation examines how PWA participate in interactive exchanges, manage turn‐taking, and repair breakdowns during naturalistic conversations. Furthermore, the Verbal Activity Log acknowledges the various modes through which PWA can express themselves and interact with others, thus offering a multidimensional view of their communicative abilities. Noteworthy, these measures assist in the evaluation of a person's practical language ability and their capacity to engage in meaningful, everyday conversations.

#### 3.3.4 Complexity of Language

Assessment of informativeness and complexity provides a measurable framework to determine efficient, meaningful, and effective communication for both the PWA and their communication partners. Assessments included in our study that evaluate this subcomponent include Correct Information Units (CIU) (Nicholas and Brookshire 1993), the Communication Activities Checklist (Cruice 2002), and Informational Units (McNeil et al. 2001). This subcomponent, primarily reflecting the Body Structures and Functions (Impairments) component of the WHO‐ICF model (WHO 2001), may be important in the enhancement of the individual's ability to express themselves clearly and succinctly, which, in turn, will promote improved functional communication in daily life. This heightened ability may translate to improved functional communication in various real‐life situations, providing a more comprehensive and accurate evaluation of their communicative competence within specific contexts. These assessments recognize the importance of multiple communicative channels: verbal, non‐verbal, written, and possibly the use of alternative communication systems to evaluate the informativeness and complexity of communication. Informational Units specifically incorporate interactiveness by assessing the exchange of information in a dialogue context. The Communication Activities Checklist addresses the complexity of language across various daily tasks, capturing the informativeness and intricacy of their expressive abilities. This not only includes the content of the communication but also the effectiveness of the interaction between PWA and their conversational partners. Such an approach acknowledges the real‐world challenges and scenarios faced by PWA and aids in developing strategies to enhance their ability to engage in meaningful, reciprocal communication.

#### 3.3.5 Naturalistic Scenarios

Analysing communication in day‐to‐day contexts and conversation helps identify barriers and challenges that PWA face in real time. It may also guide the development of strategies to overcome these communicative hurdles and improve overall communication. Assessments such as Transactional Success (Ramsberger and Rende 2002), the Social Activities Checklist‐2 (Cruice 2012), and the Communication Effectiveness Index (Lomas et al. 1989) aim to determine the level of effective communication and overall achievement of successful interactions. Transactional Success employs a multimodal approach by assessing not just verbal communication but also non‐verbal cues and written abilities, thus providing a comprehensive understanding of a PWA's communicative capabilities. The Transactional Success framework further incorporates interactiveness by analysing the give‐and‐take nature of conversations, examining how individuals navigate and negotiate meaning in real‐time exchanges. The Social Activities Checklist focuses on naturalistic scenarios by assessing how individuals participate in social activities and group settings. Similarly, the CETI emphasizes interactive communication by considering the context in which communication takes place, thus recognizing the dynamic and fluid nature of everyday interactions.

### 3.4 Assessment Tools Not Specific to Aphasia

Two prominent tools that we identified in our search have broader applicability across multiple communication disorders, without specifically focusing on aphasia. However, the Pragmatics Profile (Dewart and Summers 1996) and Goal Attainment Scaling (GAS; Kiresuk and Sherman 1968) both assess functional communication from different perspectives (Dewart and Summers 1996; Kiresuk and Sherman 1968) and may offer important insights into social interaction, pragmatic skills, and individualized goal achievement.

#### 3.4.1 Pragmatics Profile

The Pragmatics Profile of Everyday Communication Skills is a tool designed to assess the pragmatic aspects of communication, which refers to how language is used in social interactions (Dewart and Summers 1996). Unlike assessments that focus solely on linguistic abilities, the Pragmatics Profile takes a broader view by examining how individuals initiate, maintain, and conclude conversations, as well as how they use non‐verbal communication such as gestures, facial expressions, and eye contact. It also assesses how well individuals adapt their communication style to different contexts, such as formal versus informal settings. The Pragmatics Profile is typically conducted through interviews with caregivers, teachers, or individuals who know the person well. These informants provide insights into the individual's communication behaviour across various everyday situations. Additionally, observations may be used to gather data on how the person communicates in real‐world settings. For PWA, the Pragmatics Profile may be particularly useful in assessing how they cope with the social aspects of communication, such as turn‐taking in conversation and using non‐verbal cues to supplement spoken language. The assessment relies on subjective reports from informants, which can introduce variability and potential bias. Furthermore, because it does not involve direct testing of communication skills, it may miss some subtleties in the individual's communication abilities that would be evident in a more structured testing environment.

#### 3.4.2 GAS

GAS is a personalized assessment method widely used in clinical, therapeutic, and rehabilitation settings. The strength of GAS lies in its ability to measure an individual's progress toward achieving specific, tailored goals. The measure is particularly useful in cases where standardized assessments may not capture the unique, individualized outcomes that are important for the person being assessed. In the context of speech‐language therapy, GAS is often used to track progress toward communication‐related goals (Kiresuk and Sherman 1968). An individual's goal might be to improve their ability to use gestures to supplement verbal communication or to participate in social conversations more effectively. Each goal is scaled on a 5‐point continuum, with scores ranging from −2 (much less than the expected outcome) to +2 (much more than the expected outcome). This flexible framework allows for highly individualized goals to be set, whether they focus on verbal communication, non‐verbal communication, or the use of AAC strategies. One of the key strengths of GAS is its flexibility, as it adapts to each individual's specific needs, providing a clear measurement of progress toward goals that are meaningful to them. The subjectivity involved in setting and rating goals can lead to variability in how progress is interpreted, depending on the SLP's judgements. Additionally, the process can be time‐consuming, as it requires careful goal setting, scaling, and follow‐up assessment.

## 4 Discussion

The findings of this review reveal that functional communication assessments vary significantly in how they address the criteria of contextuality, multimodality, and interactiveness even when these criteria are explicitly included. While assessments may meet one or more of these criteria, the extent to which they fulfil each varies, impacting their applicability in different clinical and research contexts.

### 4.1 Variability in Contextuality

Some tools, such as the IFCI, are highly contextual because they evaluate communication in real‐world, naturalistic settings, such as hospitals, where patients communicate their specific healthcare needs. This approach directly reflects functional communication as it occurs in the participant's actual environment. By contrast, assessments like the CADL‐3 simulate real‐life scenarios, such as making a phone call or reading a grocery list. While these tasks provide some contextual relevance, they lack the environmental and situational dynamics present in naturalistic settings. Tools like the Aphasia Impact Questionnaire (AIQ) further expand contextuality by asking participants to reflect on their communication experiences in everyday life, such as shopping or interacting with others. However, the AIQ relies on recollection rather than direct observation or testing in these contexts. These distinctions demonstrate the need to differentiate between simulated contexts, recollection‐based contexts, and direct, real‐world contexts in functional communication assessments.

### 4.2 Variability in Multimodality

Multimodality, or the integration of multiple communication modes, is another area where assessments differ. In our framework, multimodality is not only encouraged but explicitly assessed, with each modality evaluated for both its use and effectiveness. Simply allowing individuals to use multiple communication modes is insufficient; assessing them separately provides critical insights into how different modalities contribute to overall communicative success and informs targeted intervention strategies. For example, the ASHA Functional Assessment of Communication Skills (ASHA‐FACS) explicitly evaluates verbal, non‐verbal, and AAC modes, offering a comprehensive multimodal perspective. Similarly, the Scenario Test incorporates gestures and AAC strategies alongside verbal communication, reflecting a dynamic, multimodal approach to functional communication. In contrast, assessments like the ANELT and CIU focus primarily on verbal communication and do not explicitly evaluate non‐verbal or AAC strategies. While these tools may implicitly involve multimodal elements, their primary emphasis remains on spoken language. By comparison, tools like the AAC Categories of Communicators Checklist exclusively focus on AAC use but do not address the integration of verbal or non‐verbal modes.

### 4.3 Variability in Interactiveness

Assessments categorized as interactive capture dynamic, two‐way exchanges, yet they differ in the depth and nature of this interaction. For example, the Inpatient Functional Communication Interview (IFCI) evaluates reciprocal exchanges specifically within real‐world healthcare interactions, such as communication between patients and medical staff in hospital settings. This assessment involves structured but adaptive interactions where the examiner responds in real‐time to the individual's communication attempts, incorporating natural turn‐taking and repair strategies that occur in clinical environments.

In contrast, the Communication Activities of Daily Living (CADL‐3) includes interactive role‐play tasks, but these are pre‐scripted and standardized, meaning that the examiner's responses are predetermined rather than dynamically adjusted based on the individual's communicative behaviour. As a result, while both tools involve role‐playing, the IFCI better approximates real‐world communicative demands by allowing for spontaneous variation in the interaction, whereas the CADL‐3 provides a controlled, structured simulation that may not capture the full range of adaptive communicative strategies used in everyday life.

Similarly, tools like the Communicative Effectiveness Index (CETI) rely on caregiver‐reported observations of interactive communication but do not involve live, dynamic exchanges, thereby offering an indirect perspective on interactivity. While assessments may be classified as interactive, the nature of interaction ranges from real‐world, naturalistic exchanges to structured simulations or observational frameworks.

### 4.4 Assessment Tools Not Specific to Aphasia

While neither the Pragmatics Profile nor GAS were originally designed for PWA, they provide essential elements that could strengthen the construction of a well‐rounded functional communication assessment for PWA. Incorporating pragmatic and goal‐based approaches would ensure that assessments not only capture deficits but also measure adaptive communication strategies, real‐world interactions, and meaningful progress. Future work should explore how these broader frameworks can be tailored to aphasia‐specific needs, ultimately leading to more ecologically valid assessments and person‐centred treatment planning.

### 4.5 General Discussion

We have explored both formal and informal assessment measures of functional communication to improve our understanding of the bases on which SLPs may make clinical decisions in their evaluation of PWA, as well as to provide insights to researchers, caregivers, and PWA themselves on this important prerequisite to life participation. Informal functional communication assessments can offer unique insights and perspectives that formal assessments might not fully capture. Qualities including personalization, contextual relevance, dynamic interaction, flexibility, and cultural sensitivity reflect the importance of considering different approaches to comprehensively evaluate an individual's functional communication abilities. While formal assessments provide valuable standardized measures, recognizing and incorporating these qualities of informal assessments can improve the overall evaluation process and lead to a more comprehensive and accurate understanding of an individual's functional communication abilities. In order to support and assess recovery in PWA, SLPs must have access to relevant, comprehensive measures to improve diagnostic and treatment outcomes. For example, for a measure to be relevant, it must provide relatable scenarios for contextualization, as highlighted in the CADL‐3 and the Scenario Test. It should assess interactional communication, as demonstrated in the Scenario Test, and be inclusive across a broad spectrum of communication abilities, as seen in tools like the ANELT and the CADL‐3.

The inherent idiosyncrasies in the wide range of informal assessments of ‘functional communication’ pose a challenge to the identification of specific gaps in the research base. With informal evaluations making up almost 90% of functional communication assessments, each non‐standardized measure estimates functional language success dependent on different communication characteristics, reflecting the respective authors’ determinations of what the most relevant components of functional communication are. Even the comparison across the limited number of formal functional communication assessments reflects the absence of a universally accepted definition of functional communication.

In our detailed search, we aim to recognize challenges and identify opportunities for enhancement in functional communication assessments by promoting the need for new research that could inform standardization efforts, identify overlooked or emerging tools, update the existing knowledge base, facilitate comparison across existing tools, and contribute to the creation of a new, unified assessment framework. We seek to resolve variability in how functional communication is evaluated and understood.

While the significance of functional communication, individualized care, and a broader understanding of health has been acknowledged, practical implementation has been challenging due to factors such as limited access to resources, the availability of standardized assessment tools, as well as entrenched traditional practices that may reinforce a false dichotomy between linguistic accuracy and functional efficacy, often prioritizing the former. In a comprehensive approach in line with the WHO‐ICF model, Activities and Participation can be aided by improvements in Body Structures and Functions so that these components are not in opposition but rather go hand in hand.

With the incorporation of the ICF model's emphasis on the relationship between physical changes, personal factors, and the environment in the context of aphasia, alongside the essential guideline provided by LPAA to further enhance the rehabilitation and well‐being of PWA, we can recognize the need to apply these models to advance assessment of functional communication (Chapey et al. 2000; WHO 2001). These models place a strong emphasis on fostering communication competence and helping PWA reintegrate into their social and communicative environments, ultimately promoting a more fulfilling and meaningful life in recognition of the challenges posed by aphasia with the idea that we can focus on the interconnection between more than just one aspect of someone's life. Future research should continue to explore how assessments align with the principles of these frameworks, such as activity and participation outcomes, to further guide the development of comprehensive, patient‐centred tools.

Although we identified three informal assessments that meet all criteria (contextuality, multimodality, and interactiveness), one might ask: why not just rely on these informal tools exclusively? While these assessments provide valuable insights into functional communication in naturalistic settings, there remains a critical need for standardized measures. There are several potential barriers to the more extensive integration of functional communication into clinical practice and research. Healthcare and insurance systems typically prioritize standardized assessments because they produce quantifiable data that are easier to document and justify for reimbursement purposes. Functional communication assessments, by contrast, often involve qualitative or contextual data, which can be more difficult to translate into billing codes or insurance claims, creating financial and administrative barriers for SLPs. SLPs may feel more comfortable using traditional, well‐researched tools that focus on linguistic impairments, especially given limited time and resources in many clinical settings. Functional communication assessments, which require assessing communication across diverse, real‐world contexts, may feel less familiar or more complex to implement, particularly when training on such approaches is limited. Functional communication is inherently context‐dependent and can vary widely across individuals and settings. The subjective nature of real‐life communication makes it more challenging to create assessments that can be consistently administered and scored across different contexts. Yet, the goal of speech and language intervention is, of course, not merely to improve individuals’ speech and language in isolation but to empower PWA to fully participate in life at work, at home, in social settings, and in their communities. Functional communication assessments are about ensuring that individuals can express themselves, engage with others, and enjoy fulfilling lives, regardless of their communication difficulties. Standardization of measures that cover all aspects of functional communication will help to solidify this recognition.

Contextualization, multimodality, and interaction form the foundation for establishing adequate measurement of functional communication (Doedens and Meteyard 2020). A standardized assessment of functional communication that meets these criteria is beneficial not only for diagnostics but also for the application of functional outcomes in treatment studies, ensuring that research findings are both reliable and comparable across different studies. An appropriate and reliable formal assessment may help establish baseline data of functional communication, track changes over time, and assess the effectiveness of interventions, contributing to evidence‐based practice in clinical speech pathology and ultimately improving the quality of life for PWA.

For the objective assessment of both verbal communication effectiveness and multimodal language use, recent developments in the use of artificial intelligence in text as well as visual‐world analysis provide exciting prospects. Though outside the scope of this review, we expect that such applications will soon be part of a comprehensive assessment of functional communication that can be broadly used in research and clinical practice. Can this promote the ability to quantify subjective elements, such as meaningful gesture use, intention, emotion, and personal perception? Quantifying such subjective elements requires a delicate balance between complexity reduction and the preservation of the authenticity of individual experiences. As a shared goal to maintain the authenticity of subjective elements, we acknowledge that subjective elements represent personal aspects that withstand easy quantification. By keeping this balance in mind, we ensure that our quantitative aspects of assessment enhance rather than compromise the genuine, individual nature of subjective experiences, contributing to a more holistic understanding of the complex dimensions of functional communication and language within the evaluation process.

### 4.6 Limitations

Limitations of this review follow from the inconsistent use of the term *functional communication* in the existing literature. Without adequate rationale, it is challenging to set boundaries for which measures are to be considered ‘assessments of functional communication’. As a result, it was challenging to compare formal assessments of functional communication in the absence of a well‐established basis for assessing language in specific situations. Additionally, our review focuses exclusively on PWA, excluding other populations with communication disorders such as primary progressive aphasia, dementia, autism spectrum disorder (ASD), and other neurological conditions. Exploring how these populations promote functional communication components could also inform advancements in aphasia rehabilitation. A future literature review that encompasses all communication‐impaired populations would offer a more comprehensive overview of functional communication tools and their broader applicability across disorders, potentially enhancing assessment practices in the field of aphasia. Also, our review may be limited by the language of publication. Studies or assessments not published in English might have been excluded, which could affect the comprehensiveness of the review, particularly if relevant research exists in non‐English‐speaking countries.

## 5 Conclusion

The purpose of this scoping review was to provide a comparative evaluation of functional communication assessments, focusing on their alignment with the criteria of contextuality, multimodality, and interactiveness. Our findings highlight the diversity of tools available, including both formal (standardized) and informal (non‐standardized) assessments, as well as their varying strengths in addressing these three criteria. While no single assessment fully integrates contextuality, multimodality, and interactiveness, many tools offer valuable insights into specific aspects of functional communication.

It is important to acknowledge that the perceived gaps in comprehensive assessment tools may reflect the framework used in this review to evaluate assessments rather than a complete lack of tools addressing functional communication. Our focus was on identifying how assessments align with dynamic, real‐world communication demands, rather than evaluating their broader availability or psychometric properties. By applying these criteria, this review aims to contribute to the ongoing conversation about the need for functional communication tools that better capture the complexities of everyday interactions.

We focus on the need for assessments that integrate multiple criteria to better reflect the complex, real‐world nature of functional communication. In our scoping review, we identified 32 functional communication assessments designed for individuals with aphasia, comprising 28 informal assessments and 4 formal assessments. Future efforts should prioritize the development of tools that are contextual, multimodal, and interactive, while aligning with frameworks that emphasize activity and participation in everyday life. By doing so, functional communication assessments can continue to evolve to meet the needs of diverse populations, including PWA.

## Data Availability

This article is a scoping review and does not include any original data. No datasets were generated or analysed during the current study.
